# Tomato *ACS4* is necessary for timely start of and progression through the climacteric phase of fruit ripening

**DOI:** 10.3389/fpls.2014.00466

**Published:** 2014-09-16

**Authors:** Suzanne W. Hoogstrate, Lambertus J. A. van Bussel, Simona M. Cristescu, Eric Cator, Celestina Mariani, Wim H. Vriezen, Ivo Rieu

**Affiliations:** ^1^Department of Molecular Plant Physiology, Institute for Water and Wetland Research, Radboud UniversityNijmegen, Netherlands; ^2^Department of Molecular and Laser Physics, Institute for Molecules and Materials, Radboud UniversityNijmegen, Netherlands; ^3^Department of Applied Stochastics, Institute for Mathematics, Astrophysics and Particle Physics, Radboud UniversityNijmegen, Netherlands; ^4^Molecular Breeding, Bayer Crop Science Vegetable SeedsNunhem, Netherlands

**Keywords:** *ACS2*, *ACS4*, climacteric fruit ripening, ethylene, *RIN*, tomato

## Abstract

Climacteric fruit ripening, as it occurs in many fruit crops, depends on a rapid, autocatalytic increase in ethylene production. This agriculturally important process has been studied extensively, with tomato simultaneously acting both as a model species and target crop for modification. In tomato, the ethylene biosynthetic genes *ACC SYNTHASE2* (*ACS2*) and *ACS4* are highly expressed during fruit ripening, with a combined loss of both *ACS2* and *ACS4* activity preventing generation of the ethylene burst necessary for fruit ripening. However, the individual roles and importance of *ACS2* and *ACS4* have not been determined. In this study, we examined specifically the role of *ACS4* by comparing the phenotype of an *acs4* mutant firstly with that of the wild-type, and secondly with two novel *ripening-inhibitor* (*rin*) mutants. Ethylene production during ripening was significantly reduced in both *acs4-1*, and *rin* lines, with *rin* genotypes showing the weaker ethylene burst. Also i) the time between anthesis and the start of fruit ripening and ii) the time required to progress through ripening were significantly longer in *acs4-1* than in the wild type, but shorter than in the strongest *rin* mutant. The delay in ripening was reflected in the lower expression of ripening-related transcripts during the mature green and light red ripening stages. Furthermore, expression of *ACS2* and *ACS4* was strongly dependent on a functional *RIN* gene, while *ACS2* expression was largely independent of *ACS4*. Altogether, we show that *ACS4* is necessary for normal progression of tomato fruit ripening and that mutation of this gene may provide a useful means for altering ripening traits.

## Introduction

Climacteric fruit ripening is a mechanism by which fully grown fruits go through a final phase of changes in texture, color, smell, and taste. It is accompanied by a burst in respiration and a rapid, autocatalytic increase in the production of the gaseous plant hormone ethylene (Alexander and Grierson, 2002). Because of the importance of climacteric fruits for the human diet (e.g., tomato, apple, banana, mango, avocado, and passion fruit) this type of ripening has been studied extensively, with tomato (*Solanum lycopersicum*) serving as both a model species and target crop for modification.

Research on non-ripening tomato mutants has provided detailed insights into the regulation of climacteric ripening. One of the best studied of these tomato mutants is *ripening-inhibitor (rin)*, in which virtually all measured ripening phenomena, like the ethylene burst, change in color, fruit softening and the production of flavor compounds are inhibited (Tigchelaar et al., 1978). The dramatic phenotypic effect of the *rin* mutant suggests that RIN is a master regulator for many ripening related processes. *RIN*/*rin* hybrids are widely used in commercial tomato production because of their extended shelf-life (Vrebalov et al., 2002). Exogenous ethylene can still activate ethylene response genes in a *rin* background, but less effectively than in the wild type (Lincoln and Fischer, 1988), suggesting that RIN also has ethylene-independent functions and thus acts upstream of both ethylene- and non-ethylene-mediated ripening processes. In 2002, Vrebalov et al. showed that disruption of the gene *LeMADS-RIN* is responsible for the ripening phenotype of the *rin* mutant (Vrebalov et al., 2002). RIN is part of the MADS-box transcription factor family, known to function as DNA-binding protein dimers consisting of two interacting MADS monomers (Ng and Yanofsky, 2001; Ito et al., 2008; Smaczniak et al., 2012). Other well studied genes involved in the regulation of tomato fruit ripening are *COLORLESS NONRIPENING* (*CNR*), an SBP-type transcription factor (Manning et al., 2006) and *NONRIPENING* (*NOR*), a NAC transcription factor (Seymour et al., 2013). Complex interactions exist between *RIN*, *CNR*, and *NOR*, with *NOR* acting upstream and downstream *RIN* (Martel et al., 2011; Osorio et al., 2011) and *CNR* being both a target of RIN as well as a necessary factor for RIN DNA binding activity (Martel et al., 2011). Furthermore, RIN probably acts as part of a protein complex containing another ripening-associated MADS-box protein, TAGL1 (Giovannoni, 2007; Vrebalov et al., 2009).

RIN has been shown to directly interact with promoters of genes involved in the major pathways associated with ripening, like ethylene biosynthesis, ethylene perception, downstream ethylene response, cell wall metabolism, and carotenoid biosynthesis (Fujisawa et al., 2011, 2012; Martel et al., 2011). The induction of ethylene biosynthesis is an essential process in the ripening of climatic fruit (Alexander and Grierson, 2002). Ethylene is synthesized from methionine, which is converted to S-adenosyl-L-methionine in the Yang-cycle (Adams and Yang, 1979). S-adenosyl-L-methionine is processed into 1-aminocyclopropane-1-carboxylic acid (ACC) by ACC synthase (ACS) and subsequently the ACC is oxidized by ACC oxidases (ACO) to form ethylene (Kende, 1993 and references therein). Because ethylene is involved in a wide range of developmental processes throughout the plant, ethylene synthesis, perception and signaling must be tightly controlled (Lin et al., 2009). There are at least 12 *ACS* and 7 *ACO* genes in the tomato genome with specific temporal and spatial expression patterns (Seymour et al., 2013). It is suggested that the production of ethylene is regulated by two different systems (McMurchie et al., 1972). System 1 is pre-climacterically active and is responsible for providing the basal levels of ethylene which are found in developing fruits and vegetative tissues. *ACS1a* and *ACS6* are thought to fulfill the function of ACC synthase in the auto-inhibitory ethylene synthesis of system 1 (Zarembinski and Theologis, 1994; Oetiker et al., 1997; Barry et al., 2000). When the fruit has reached a stage at which it is ripening-competent it goes through a transitional phase in which the expression of *ACS1a* is temporarily enhanced and the expression of *ACS4* is induced. The RIN protein is thought to play a role during this transitional phase via the direct transcriptional activation of *ACS2* and *ACS4* (Barry et al., 2000; Fujisawa et al., 2011, 2012; Martel et al., 2011). Higher concentrations of ethylene trigger system 2, the system active during climacteric ripening of fruit and characterized by an increase in the expression of *ACS2* and *ACS4*. Upon activation of system 2, the expression of *ACS1a* and *ACS6* decreases and the ethylene production becomes auto-stimulatory (Barry et al., 2000). Consequently, the hyper-activation of *ACS2* and *ACS4* expression causes a burst of ethylene. This big increase in ethylene production promotes the ripening process and is essential for normal completion of ripening in climacteric-ripening plants.

Tomato plants expressing an antisense gene targeting *ACS2* show strong down regulation of both *ACS2* and *ACS4*. The antisense fruit slowly develop an orange color but never turn red and soft nor develop an aroma (Oeller et al., 1991). They have reduced ripening-related ethylene synthesis, down to 0.1% of control fruit, and show no respiratory burst. Treatment of the antisense plants with exogenous ethylene rescues the phenotype, induces the respiratory burst and initiates the ripening process (Oeller et al., 1991). These plants show that down-regulating *ACS2* and *ACS4* has a strong effect on ethylene production and ripening. However, the individual roles and importance of these genes have not been determined. In this study, we examined a newly generated *acs4* mutant with a homozygous mutation introducing a stop-codon in the first half of the ACS4 coding sequence. We studied the effect of the mutation on ethylene production of the fruit, ripening timing and the expression of ripening-related genes.

## Materials and methods

### Plant material

*Solanum lycopersicum* var. TPAADASU was used for all experiments. For the ethylene measurements, plants were grown under standard greenhouse conditions, with 16 h/day and 8 h/night (assimilation lights used during the day at less than 250 W/m^2^) and temperature kept above 20/18°C (day/night). For the experiment regarding ripening time and gene expression analysis, plants were grown under standard greenhouse conditions, with 16 h/day and 8 h/night (assimilation lights used during the day at less than 300–350 W/m^2^) and temperature kept above 20.5/15.5°C (day/night). Genotypes were randomly distributed in the greenhouse. Self-pollination was stimulated by artificial vibration of flower clusters three times per week to get homogeneous fertilization and fruit size. Fruit stages were based on color, as determined by a single person throughout the experiments. Used stages were: mature green (MG), turning (T), orange (O), light red (LR) and red (R) (Gillaspy et al., 1993; Steinhauser et al., 2010). Because the coloring of the different lines was not uniform, the turning stage represented the fruits that were halfway in between mature green and orange. This included fruits that had a green skin with marks of orange and fruits that were more uniformly yellow. Green fruit of the wild type were harvested on average 36 days after pollination.

### Mutant generation and identification

Mutations were identified by screening a TPAADASU EMS M2 mutant population for two PCR fragments with the TILLING protocol adapted for a HRM LightScanner platform as described previously (Gady et al., 2009). The *acs4-1* mutant presented here was identified in a gene fragment amplified using the following primers: 5′-GCTATCGAAGAGGCCTATGAAAAAGG-3′ (forward) and 5′-CACAAATTCATCGTCAGACAACATG-3′ (reverse). Both mutations in the *RIN* gene were identified in a PCR fragment generated with primers 5′-TTGATGAAATTGATTTTCTTGTTG-3′ (forward) and 5′-AGCAAGTTGATCAAGAATGTGTT-3′ (reverse). PCRs were performed as follows on fourfold genomic DNA pools and, after positive pool selection, on single family genomic DNA: 94°C for 2 min; 40 cycles of 94°C for 5 s, 68°C for 10 s, and 72°C for 10 s; a final denaturation step of 30 s at 94°C; and renaturation by cooling to 30°C. RT-PCR to analyze *rin-3* transcript length was done with a standard PCR program, using primers 5′-CCAAGACATTGGAGAGATACCA-3′ (forward) and 5′-TTTGCCTCAATGATGAATCC-3′ (reverse).

### Ethylene measurements

Three individuals per genotype were used. After harvesting, fruits were left to recover for 1 h (for the stages T, O and LR) or 3 h (for stage MG) before being weighted and placed into glass cuvettes connected on-line with a laser-based ethylene detector (ETD-300, Sensor Sense B.V. Nijmegen, the Netherlands) (Cristescu et al., 2008). For mature green fruit the air flow was set at 1 L/h, for all other stages at 4 L/h. Ethylene measurement was performed as described before (Nitsch et al., 2012). The samples were measured at least twice for 10–12 min and a representative measurement was selected. Four to six biological replicas were used per stage and line. The ethylene emission was corrected for flow, weight, and background ethylene in the air.

### Ripening time measurements

For the mutant *rin-3*, two individuals were used; for the wild type and the *rin-2* and *acs4-1* mutant lines five. On specific time points during a period of 4 weeks the stage of each individual fruit (MG, T, O, LR and R) was determined. Based on these observations a model was developed to estimate the effect of the genotype on the number of days between anthesis and the start of ripening as well as on the ripening time (i.e., number of days between the first signs of ripening and the red stage) for the different plant lines in this study. As covariates the plant number, which truss the fruit was on and the fruit number were considered. The data was censored, since it was not known which day the fruit entered in a particular phase, only the phase at a fixed number of time points was known. Since this limited the amount of information in the data, not all possible interactions were considered. Furthermore, some plants carried very few fruits at the end of the experiment; the model therefore included a random effect for the plant number. *Y* = (*Y*^(*1*)^, *Y*^(*2*)^)^*t*^ was defined as the two time points at which the plant entered the two stages of interest (i.e., first sign of ripening and actual ripening), *p* as an index indicating the plant number, *b* as an index indicating the truss and finally *f* as an index indicating the fruit number, forming the following model:

$$\left(\begin{array}{c} Y_{pbf}^{\left(1\right)}\\ Y_{pbf}^{\left(2\right)} \end{array}\right)=\left(\begin{array}{c} \mu^{\left(1\right)}+\alpha_{m(p)}^{\left(1\right)}+\beta_{b}^{\left(1\right)}+\gamma_{f}^{\left(1\right)}\\ \mu^{\left(2\right)}+\alpha_{m(p)}^{\left(2\right)}+\beta_{b}^{\left(2\right)}+\gamma_{f}^{\left(2\right)} \end{array}\right)+Z_{p}+U_{pbf}.$$

Here, *m(p)* is the genetic modification of plant *p*, while μ, α_*m*(*p*)_, β_*b*_, and γ_*f*_ are parameters of the linear model, and *Z_*p*_* is a random effect corresponding to each plant, such that

$$Z_{p}\sim N_{2}\;\left(\left(\begin{array}{c} 0\\ 0 \end{array}\right),\;\sum_{0}\right),$$

where Σ_0_ is an unknown 2-by-2 covariance matrix. Finally, *U*_*mpbf*_ is an independent random fluctuation

$$U_{pbf}\sim N_{2}\left(\left(\begin{array}{c} 0\\ 0 \end{array}\right),\;\sum \right),$$

with Σ unknown. Due to the censoring of the data *L*^(*i*)^ = *L*^(*i*)^_*mpbf*_ and *R*^(*i*)^ = *R*^(*i*)^_*mpbf*_ are defined as the last time the fruit was observed before, respectively after the relevant phase started (i C; *L* ≤ *Y* ≤ *R*). The log-likelihood of all observations as a function of the parameters is given by:

$$\begin{array}{l} \sum_{p=1}^{26}\log({𝔼}_{\sum_{0}}(\prod_{b,f}{\mathbb{P}}_{\sum }(U+\mu +\alpha_{m(p)}+\beta_{b}+\gamma_{f}\\ \;+Z\in \left[L_{pbf}^{\left(1\right)},R_{pbf}^{\left(1\right)}\right]\times \left[L_{pbf}^{\left(2\right)},R_{pbf}^{\left(2\right)}\right]))), \end{array}$$

Where *U* under P_σ_ has a N(0, Σ)-distribution and *Z* under *E*_σ0_ has a N(0, Σ_0_)-distribution. This model was used on the data of 26 plants (including 9 plants from non-presented genotypes) and a full factorial design with respect to the truss, the fruit and the plant. Maximum likelihood was applied to estimate the parameters of this model.

### Real-time quantitative RT-PCR analysis

Fruits were obtained from at least two individuals per genotype. Directly after harvesting the fruit, the pericarp tissue was collected, cut into small pieces, frozen in liquid nitrogen and stored at −80°C. The pieces were ground into powder with piston and mortar in liquid nitrogen to keep the tissue frozen. 200 mg of powder was used to isolate total RNA (RNeasy Plant Mini kit, Qiagen). The RNA was treated with DNase (TURBO DNA-*free*, Ambion) and 0.9 μ g was used for reverse transcription (iScript cDNA Synthesis kit, BioRad). The cDNA equivalent of 20 ng of total RNA was used in a 25 μ l PCR reaction on a thermocycler (CFX iCycler, BioRad) with cybergreen (iQ SYBR Green Supermix, BioRad). Three to four biological replicas of each sample type were analyzed with two technical replicates. Absence of genomic DNA and primer dimers was confirmed by analysis of RT-minus and water control samples and by examination of dissociation curves. PCR primers were designed using a computer program (Beacon Designer 7.51) and are listed in Supplemental Table 1. Quantitative gene expression data was analyzed according to Rieu and Powers (2009). To normalize the qPCR data, five reference genes were used (*EF-1α*, *Actin2-7*, *RPL8*, *UBQ11*, and *GAPDH3*) and the stability of these reference genes across samples was confirmed using geNORM software (V_5/6_ = 0.15; Vandesompele et al., 2002). Primer pair efficiencies were estimated by analysis of the amplification curves with LinReg v12.1 software (Ramakers et al., 2003) and the average efficiency of all reactions on a plate was used in calculations (Cook et al., 2004).

### Statistical analysis

Ethylene evolution data of the different genotypes was compared using one-way ANOVA with LSD on log-transformed data. To test hypothesis on differences in ripening time parameters, likelihood ratio testing (with asymptotic χ^2^-distributions) was applied to the ripening model described above. For analysis of the qPCR results, log-transformed normalized relative quantities were compared using a one-way ANOVA with LSD. In case of the LR stage, two datasets were combined following mean centering.

## Results

### Generation and identification of new alleles of ACS4 and RIN

**Figure 1:**
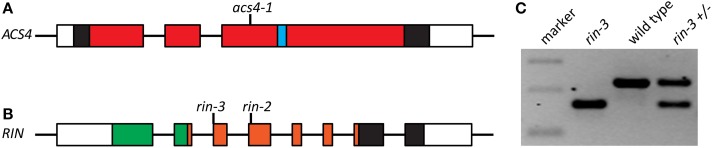
**Gene models and mutant description**. Gene models of *ACS4* **(A)** and *RIN* **(B)**, indicating the sites of mutations in the alleles presented in this study. Exons are indicated as boxes and depicted in relative sizes, introns not. Coding sequence is indicated as filled boxes; red: the canonical aminotransferase class I/classII domain (IPR004839); blue: the cofactor binding site (IPR004838); green: MADS-box domain (IPR002100); orange: K-box (IPR002487). **(C)** RT-PCR showing reduced length of the *rin-3* transcript. The 100-, 200- and 300-bp bands of the DNA marker are shown.

Using the TILLING strategy (Gady et al., 2009), a new allele of *ACS4*, named *acs4-1*, was identified in tomato. The allele had an A-to-T mutation at bp 610 of the open-reading frame resulting in a premature stop codon [K^204^ (AAA) to stop (TAA)] and a truncated protein (Figure 1A). The truncated protein is likely to represent a null-allele, as it ends in the middle of the canonical aminotransferase class I/classII domain (IPR004839, aa 25-439), before the cofactor binding site (IPR004838, aa 279-292). In the same way, two novel *RIN* alleles were identified and named *rin-2* and *rin-3*. The *rin-2* mutation results in an amino-acid substitution Leu^112^ (CTA) to Pro^112^ (CCA) in the conserved K-box (IPR002487), which promotes protein dimerization. The mutation in *rin-3* is a substitution of the last nucleotide (G to A) of intron 2. This nucleotide is part of the splicing acceptor site and cDNA sequencing showed that the mutation leads to an mRNA that misses the 62-bp long exon 3 (Figures 1B,C). This, in turn, introduces a stop codon in the ORF of the mutated gene, five codons downstream of Q^88^. The truncated protein still contains the complete MADS-box domain (IPR002100) for DNA binding, but has lost most of the K-box, making it a likely null-allele. These two new *rin* mutant lines are unique in that they are solely mutated in the *LeMADS-RIN* locus and therefore differ from the classical *rin* mutant, in which the mutation consists of a deletion of both *LeMADS-RIN* and the neighboring *LeMADS-MACROCALYX (LeMADS-MC)*, the latter being is associated with sepal development (Vrebalov et al., 2002).

### Ethylene emission

**Figure 2:**
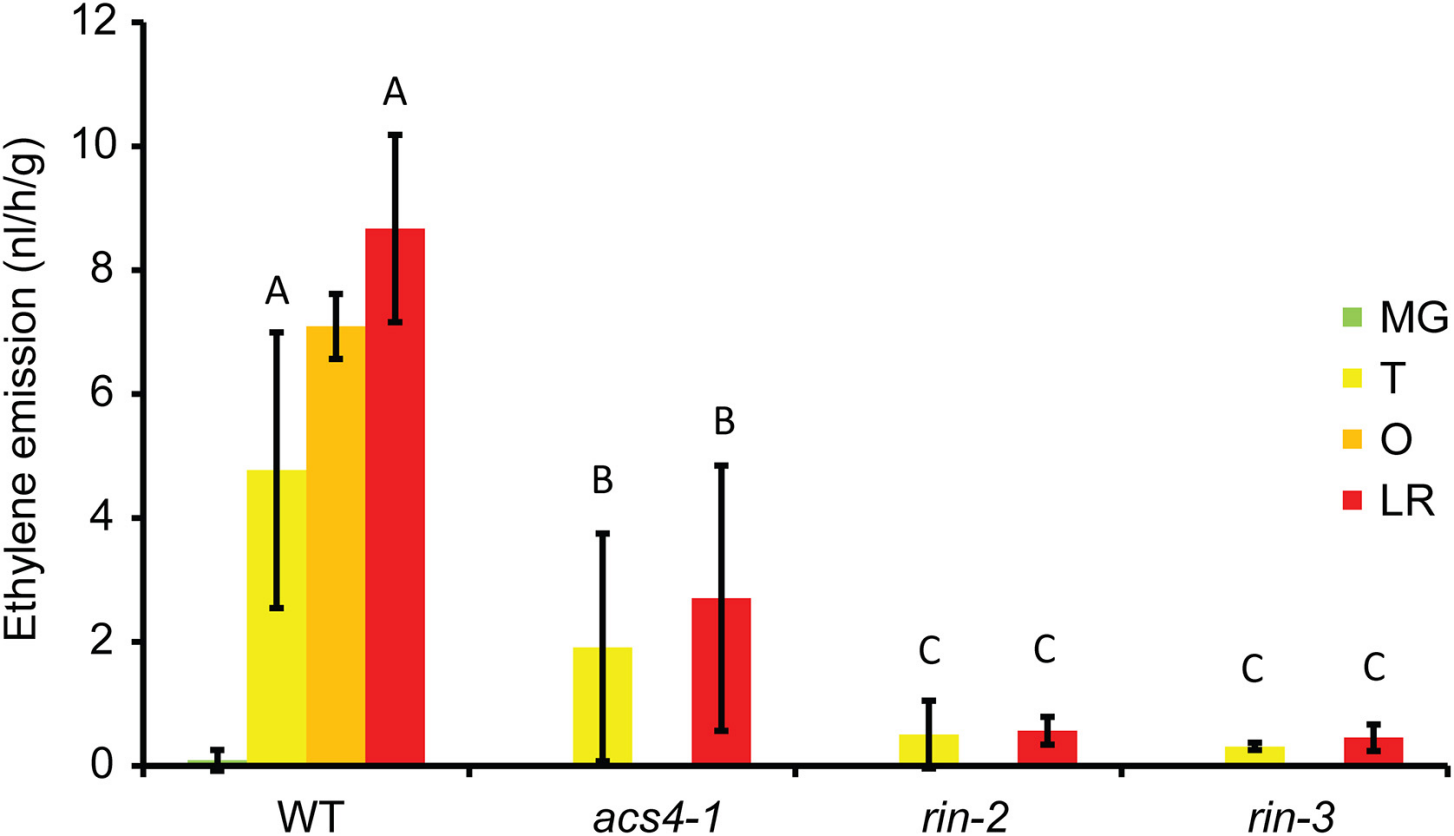
**Ethylene emission from fruits**. The graph shows ethylene emission levels at four ripening stages in the wild-type and at two ripening stages in the mutants. Values represent the average of 3 plants (4–6 fruits per plant), ±*SD*. For statistical analysis, ethylene emissions were compared among the same developmental stage, with different letter indicating a significant difference of at least *P* < 0.05.

As a characteristic of the climacteric fruit ripening process, ethylene production was strongly enhanced during the ripening phase in the wild-type tomato fruits (Figure 2). To determine the effect of the new mutations in *ACS4* and *RIN* on the production of ethylene in the fruit, ethylene emission was measured at two ripening stages, i.e., turning and light red (Figure 2). The *rin-2* and -*3* mutant fruits showed a 90–95% reduction in ethylene emission at the analyzed stages, as compared to the similar stage in the wild-type (*P* < 0.001), confirming that both alleles have severely reduced RIN function, but produced more ethylene than mature-green wild-type fruit (*P* < 0.001). *acs4-1* produced about a third to a quarter of the ethylene of the wild type at the turning and light red stages, respectively (*P* < 0.05).

### Ripening times

**Figure 3:**
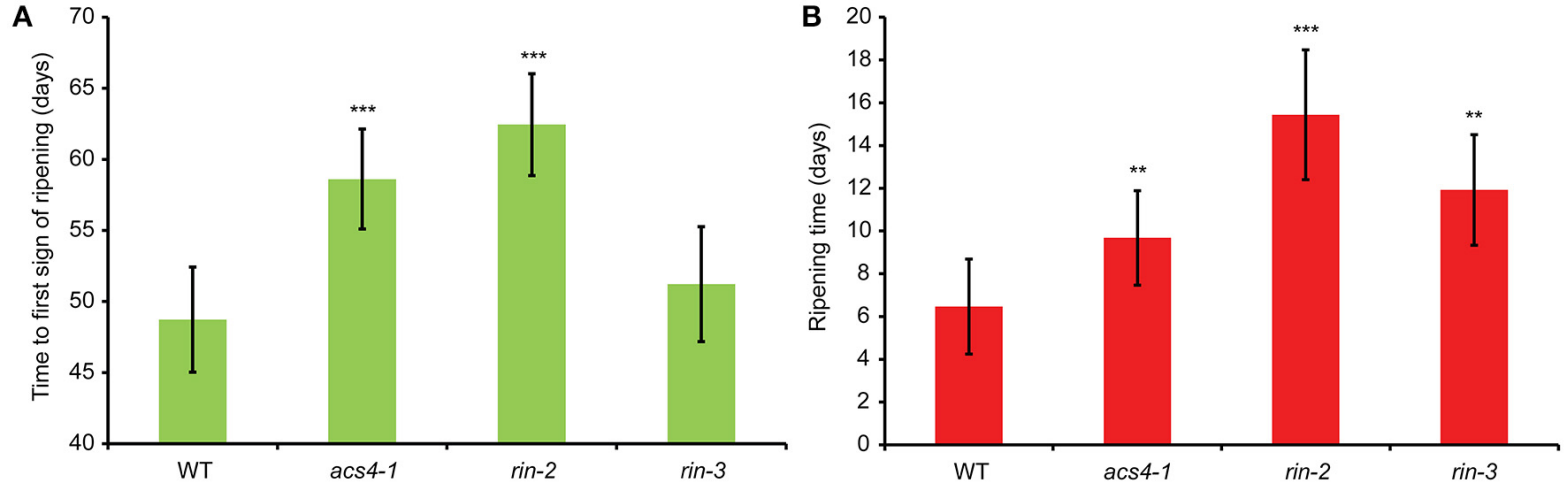
**Fruit ripening times. (A)** Estimation of the number of days from open flower until the first sign of ripening, ±*SD* (*n* = 24–60 fruits, i.e., 10–12 fruits from 2–5 plants per genotype). **(B)** Estimation of the length of the climacteric ripening phase (ripening time), ±*SD* (*n* = 28–30 fruits, i.e., 5–6 fruits from 5 plants per genotype each). ^**^, significantly different from the same stage in the wild type, *P* < 0.01; ^***^,*P* < 0.001.

To estimate the effect of the genotype on both the period before the ripening process is initiated and the time required to progress through ripening, we developed a model, taking as covariates (i) the plant individual, (ii) the position of the truss on the plant and (iii) the position of the fruit in the truss (see Materials and Methods). Likelihood ratio tests showed that the position of the truss was not a significant covariate, and therefore this factor was removed from the equation. There was an effect of the position of the fruit, where the fruits formed earlier on a truss (closer to the stem) ripened somewhat faster. Using the model we found that the *rin-2* mutant line showed a significant increase in both, the average time until fruit ripening started and the length of the climacteric ripening phase, as compared to the wild type (Figures 3A,B, also see Supplemental Table 2). The *rin-3* mutant also had an increased ripening time but showed no significant delay in the appearance of the first sign of ripening. The *acs4-1* mutant was delayed in both processes, but less so than the *rin-2* mutant.

### Expression of ripening related genes

**Figure 4:**
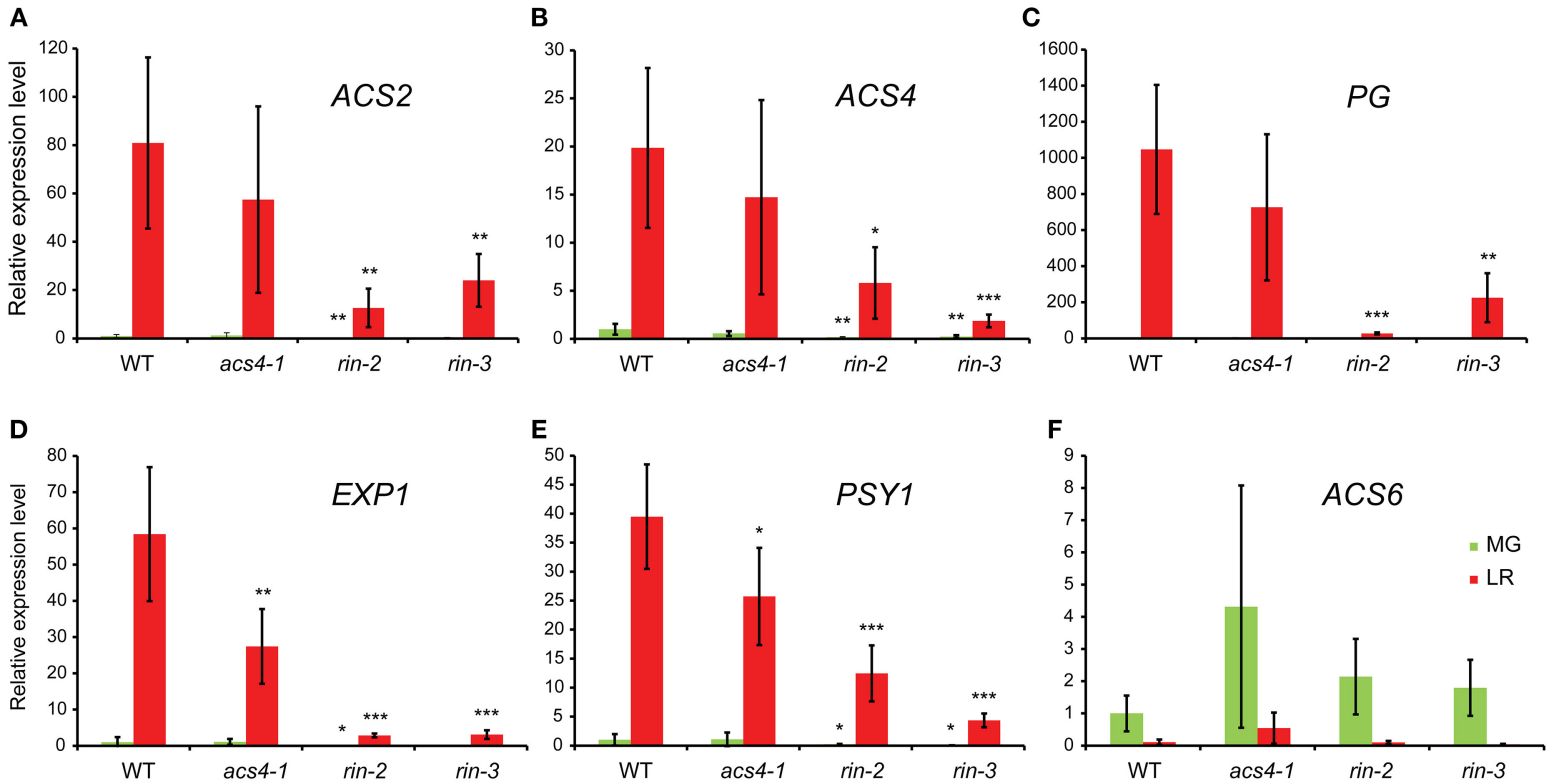
**Relative expression levels of ripening related genes in wild-type and mutant fruits**. **(A)** *ACS2*; **(B)** *ACS4*; **(C)** *PG*; **(D)** *EXP1*; **(E)** *PSY1*; **(F)** *ACS6*. In all cases, the expression level of the gene in the wild type genotype in the green stage has been valued 1. Values represent the average of 3–6 biological replicates, ±*SD*. ^*^significantly different from the same stage in the wild type, *P* < 0.05; ^**^P < 0.01; ^***^P < 0.001.

To determine the effect of the mutations on the expression of ripening-related genes, quantitative RT-PCR was used to measure the transcript levels of *ACS2*, *ACS4*, *expansin1* (*EXP1*), *phytoene synthase 1* (*PSY1*) and *polygalacturinase* (*PG*) in fruits before and during the ripening phase (Figure 4). Each of these genes is known to function during fruit ripening. *PSY1* is needed for the production of carotenoids (Bird et al., 1991) and *PG* and *EXP1* are involved in the softening of the fruit (cell wall metabolism) (DellaPenna et al., 1989; Rose et al., 1997). As expected, the expression of all five genes was strongly up-regulated from the green to the light red fruit stage in the wild-type, from around 18 times (*ACS4*) to almost 1000 times (*PG*). The *rin* mutants showed a significant reduction in expression of all these transcripts during fruit ripening, and in *rin-2* the expression of four out of the five genes was already down-regulated in mature green fruit. In the *acs4-1* mutant *EXP1* and *PSY1* expression was significantly lower than in the wild type in the light red fruit stage, but no differences were detected before the start of ripening. *ACS2* and *PG* showed the same trend, although not statistically significant. We also examined the system-1 *ACS* gene *ACS6*. In the wild-type, the expression of this gene was down-regulated during ripening (*P* < 0.01). Contrary to the genes mentioned above, the regulation of *ACS6* was not affected by the *rin* mutations, and also not by the *acs4-1* mutation.

## Discussion

By measuring the ethylene production, ripening timing and ripening-related gene expression in an *acs4-1* mutant and comparing it to control genotypes, we gained insight into the role of the tomato system-2 ACC synthase gene *ACS4* in climacteric fruit ripening.

As control genotypes, we used two novel *rin* alleles, both in the TPAADASU background. As described here, *rin-2* and -*3* are severely affected in all measured ripening characteristics, indicating that they have reduced RIN function. The phenotypes, however, are weaker than described for the original *rin* mutant. In the latter, *ACS2*, *ACS4*, *EXP1*, *PG*, and *PSY1* expression is not or hardly up-regulated during ripening (DellaPenna et al., 1989; Rose et al., 1997; Barry et al., 2000; Fujisawa et al., 2011, 2012; Martel et al., 2011), ethylene production does not rise above the pre-climacteric levels (i.e., about 1–2% of the orange to red stages in the wild type), even after 120 days of development, and fruits do not color beyond yellow (Robinson and Tomes, 1968; Herner and Sink, 1973; Tigchelaar et al., 1978). By contrast, significant up-regulation of the tested ripening-related transcripts was seen in *rin-2* and -*3* and fruit of the mutants still produced 5–10% of wild-type ethylene during ripening, well above pre-climacteric levels (Figure 2). Most tellingly, *rin-2* and -*3* fruit was able to complete fully the ripening process (Figure 3). As *rin* mutants are still ethylene responsive, the ripening-related gene expression and ripening progression could in theory be caused by exogenous ethylene from ethylene-producing neighboring plants during cultivation. However, because exogenous ethylene does not enhance ethylene production in *rin* (Herner and Sink, 1973), a more likely explanation might be that system-2 ethylene production is less *RIN* dependent in TPAADASU than in VF36, the original *rin* background or that the LeMADS-MC protein, which is not affected in *rin-2* and -*3*, might play a redundant role in system-2 ethylene production in the absence of functional RIN protein. Public transcriptome sequencing data (http://www.ncbi.nlm.nih.gov/Traces/sra/?study=SRP010775) show that this gene is relatively active in the fruit at breaker stage (Tomato Genome Consortium, 2012). Also, we found that expression of the system-1 gene *ACS6* was down-regulated upon ripening in *rin-2* and -*3* similar to wild type fruits, whereas Barry et al. (2000) did not see such down-regulation in *rin*. However, our results are similar to those on *rin* published in a public database (http://www.ncbi.nlm.nih.gov/Traces/sra/?study=SRP004923). These differences are likely to be due to differences in the stage analyzed. Further study is needed to clarify these aspects.

The *acs4-1* mutant, too, had a significantly lower rate of ethylene production, delayed ripening and reduced ethylene-dependent gene expression. These effects, however, were less severe than described for an *ACS2/ACS4* silenced line, which produced virtually no ethylene and did not ripen beyond the orange stage in up to 120 days (Oeller et al., 1991). Together, this shows that *ACS4* has a unique function in the production of system-2 ethylene and climacteric ripening in tomato, but that there is an additional, important role for *ACS2*. Although *ACS2* expression was suggested to depend on *ACS4* expression during the transition from the system-1 to system-2 ethylene synthesis (Barry et al., 2000), no significant reduction in its expression was found in the *acs4-1* mutant (Figure 4). Together with the finding that both *ACS4* and *ACS2* are direct targets of RIN (Martel et al., 2011), this does not support the idea that *ACS4* has an essential role in the system-1-to-2 transition. However, it should be noted that the fact that fruits were always compared at similar ripening stages, instead of at similar time points during development, dampens observed differences between genotypes.

In summary, we conclude that *ACS4* has an important role in the climacteric ripening of tomato fruit. A K^204^ truncation of the *acs4* protein leads to a significant delay in the ripening process on multiple levels. This allele, as well as the new *rin* alleles, may be useful to increase tomato shelf life and reduce post-harvest losses.

### Conflict of interest statement

Patents concerning mutations in the tomato ACS2, ACS4 and RIN genes were published under publication numbers WO2014079896 A1, WO2014049002 A1 and WO2013156204 A1, respectively (Nunhems B. V., Wim H. Vriezen et al). The authors declare that the research was conducted in the absence of any commercial or financial relationships that could be construed as a potential conflict of interest.
