# Supplementary material for: Tomato ACS4 is necessary for timely start of and progression through the climacteric phase of fruit ripening
Source: Front Plant Sci. 2014 Sep 16;5:466. doi: 10.3389/fpls.2014.00466 (PMC4165129; doi:10.3389/fpls.2014.00466)
Supplement: Supplementary file 1 [file Table1.DOCX]

**Supplementary table 1 – Sequences of primers used for qRT-PCR analysis.**

| **Gene** | **Forward primer (5’-3’)** | **Reverse primer (5’-3’)** |
| --- | --- | --- |
| *ACS2 (Solyc01g095080.2)* | CACGCCTCAATTCGTCAGTATAGC | GTTGCAGTAAGTCATTTCCTGTTCATC |
| *ACS4 (Solyc05g050010.2)* | GGAAATAATAAAATGGATTTGGAGACGAG | AACTACTGATGACTTGTAATTTGAAATCTC |
| *ACS6 (Solyc08g008100.2)* | ACTCTCCTCATTCACCTATGTCTTCTC | CATGTCCATAATTGAACGATGTGTGTAG |
| *PG (Solyc10g080210.1)* | GTTACTGTAAATGAAGCCAAAATTATCG | CGTCTTGCATTTCCACATTCAG |
| *EXP1 (Solyc06g051800.2)* | TTTGACCTCGCTATGCCTATGTTTC | CTAAGTTGAAGTAACGGAATCCATTGATG |
| *PSY1 (Solyc03g031860.2)* | ATTGAAGGAATGCGTATGGACTTGAG | AACCGTACCAGCAACATAATAACAATAAAG |
| *EF-1α*  *(Solyc06g005060.2, Solyc06g009970.2, Solyc06g009960.1, Solyc11g069700.1)* | CCTCCGTCTTCCACTTCAGGATG | GTCACAACCATACCAGGCTTGATC |
| *Actin2-7*  *(Solyc03g078400.2)* | GGACTCTGGTGATGGTGTTAG | CCGTTCAGCAGTAGTGGTG |
| *RPL8 (Solyc10g006580.2)* | GGTGTTCTGGTGATTACGCCATTG | CCAGCAACCTGACCAATCATAGC |
| *UBQ11 (Solyc07g064130.1)* | GCTCCGACACCATTGACAAC | GCAACAGACGCAACCAGAC |
| *GAPDH3 (Solyc03g111010.2)* | GCTGCCATCAAGGAGGAATCTG | AACCACATCATCTTCAGTGTAACCTAG |
